# Biological activities and compounds of *Volvariella volvacea*: A review of their dual roles in health and infection

**DOI:** 10.22034/cmm.2026.345482.1712

**Published:** 2026-02-01

**Authors:** Roman Rafael B. Manucdoc, Rich Milton R. Dulay

**Affiliations:** 1 Department of Biological Sciences, Central Luzon State University, Science City of Muñoz, Nueva Ecija, Philippines; 2 Office of University Research Development, Tarlac State University, Tarlac City, Tarlac, Philippines; 3 Center for Tropical Mushroom Research and Development, Central Luzon State University, Science City of Muñoz, Nueva Ecija, Philippines; 4 Department of Biology, College of Science, De La Salle University, Taft Avenue, Manila, Philippines

**Keywords:** Bioactivities, Biocompounds, Fungal Infection, *Volvariella volvacea*

## Abstract

**Background and Purpose:**

*Volvariella volvacea* is an edible mushroom that has been demonstrated to exhibit various biological activities and contain numerous biochemical compounds. However, it was reported to cause invasive infection in immunocompromised patients, affecting their body organs in the process. Infective nature of *V. volvacea* has not yet been fully elucidated. Although its bioactivities and compounds are considered pharmacologically beneficial, they were examined in this bifaceted review to provide a preliminary explanation for the harmful opportunistic behavior of the fungus.

**Materials and Methods:**

Published articles retrieved from Google Scholar were comprehensively consolidated, evaluated, and summarized. Specific keywords, relative to infection reports, biological activities, and compounds of *V. volvacea*, as well as infective mechanisms of some medically significant fungal pathogens, were used to collect and select studies for this review.

**Results:**

Related studies describing the mechanisms of opportunistic and true pathogenic fungal species, as well as other disease-related biological processes, were used as the basis for this review. Based on these findings, *V. volvacea* may induce systemic infection through its bioactivities and compounds. Overall, these properties may contribute to the growth, survival, proliferation, pathogenicity, and virulence of the fungus during infection. The antioxidant activity may help evade the defense mechanisms of the host, such as the induction of oxidative stress during an infection. Immunomodulatory mechanisms, through increased immunological cells and pro-inflammatory genes, may exacerbate damage within the tissues of the host. Similarly, cytotoxicity may also help worsen the fungal infection by impeding division of healthy cells. Meanwhile, the antimicrobial activity of *V. volvacea* may aid the outgrowth of both beneficial and pathogenic microorganisms within the host microbiota, resulting in harmful dysbiosis. The initiation of platelet aggregation, which is a defense mechanism against infection, may also be evaded by the fungus through its antiplatelet property. Moreover, the biological compounds of *V. volvacea*, namely phenols, flavonoids, and β-glucan, may also contribute to infection through their association with the aforementioned biological activities, in addition to their toxicological properties.

**Conclusion:**

This review may serve as a basis for future studies to provide a deeper understanding of the infective behavior of *V. volvacea*, paving the way for improved diagnosis, prognosis, and treatment strategies. In the meantime, adherence to precautionary and biosafety measures when handling *V. volvacea*, as well as maintaining overall health, is highly encouraged, especially for individuals with frequent exposure to the mushroom.

## Introduction

The diversity of Kingdom Fungi is vast, with approximately 200,000 identified species [1] out of the estimated 2.2-3.8 million overall species around the world [2]. Some of these fungi are the macroscopic mushrooms that have visible spore-bearing fruiting bodies that commonly grow on moist grounds, decaying logs, and trees. Since most of them are edible and possess nutraceutical and pharmacological benefits, efforts have been made to preserve them from the wild. Accordingly, their biological activities and bioactive components have been investigated to facilitate their efficient utilization. One of these beneficial mushrooms is *Volvariella volvacea*.

Being a saprobe, the free-gilled and pink-spored *V. volvacea* obtains its nutrients by utilizing decomposing organic materials. It undergoes six growth stages, including pinhead, tiny button, egg, elongation, and mature stages [3]. The mushroom, which belongs to the family Pluteaceae [4], is mainly characterized by a volva at the base of the stem. *V. volvacea* is widely distributed and commonly cultivated in Southeast Asia. It is extensively used in cuisines and widely grown as a food source. Moreover, it is considered easy to cultivate as it can be quickly grown using basic materials, such as paddy straw and/or other organic waste as substrates [5]. In addition, it is known to grow favorably at temperatures ranging from 25°C to 35°C [6-7]. Meanwhile, *in vitro* mycelial cultivation was recently performed by Ahlawat and Kaur [8], wherein significantly effective growth and morphologically distinct colony formation were observed on paddy straw and malt extract agar medium, respectively.

Similar to other organisms worldwide, mushrooms, like *V. volvacea*, play important roles in the environment. Not only do mushrooms have ecological functions as decomposers and symbionts of other life forms, but they are also known for their wide range of already elucidated beneficial biological activities. To date, antioxidant activity is the most reported biological activity of the mushroom [9-23]. The mushroom was also reported to display immunomodulatory [15, 21-27], cytotoxic [26, 28], antidiabetic [12, 29-32], antimicrobial [33-40], cardioprotective [12, 27, 41], and hepatoprotective [42] activities. Moreover, many studies have highlighted biological compounds screened from the fungus, chiefly phenols [10, 11, 13, 14, 16-20, 31], flavonoids [10, 14, 16, 18, 34], and β-glucan [14-15, 19].

However, despite the aforementioned beneficial properties, *V. volvacea* was reported to cause infection in immunocompromised patients. Displaying an opportunistic behavior, the fungus involved internal organs in the systemic infection it induced. The first report was documented by Salit et al. [43], where the patient presented with an invasive fungal infection in the lungs and brain, following a double umbilical cord blood transplantation. The present review also presents other reports of *V. volvacea* infections and emphasizes the biological activities and compounds of the fungus as potential contributing factors, providing a preliminary explication of the infective and opportunistic nature of the fungus.

### Reports of Volvariella volvacea infection

Reports of infection caused by *V. volvacea* are summarized in Table 1, highlighting the involved organs, clinical history, and diagnosis of the fungal infection among the patients [43-49]. Most sites of the infection were located in the upper body, specifically within the thoracic cavity, including the heart and lungs, as well as the brain in the cranial cavity. It is also noteworthy that all patients came from Asian countries where the climate generally favors the growth of *V. volvacea*. While mushrooms are often used for cuisine in these countries, there are only a few reports of exposure history to the fungus [46, 47]. It was revealed that the patients frequently smelled fresh *V. volvacea* fruiting bodies during their preparation for consumption.

**Table 1:** Summary of clinical details of patients presenting with *V. volvacea* infection

Patient information	Patient 1 [43]	Patient 2 [44]	Patient 3 [44]	Patient 4 [44]	Patient 5 [45]	Patient 6 [46]	Patient 7 [47]	Patient 8 [48]	Patient 9 [49]
Involved organs	Lungs and brain	Lung and brain	Skin, lung, and brain	Skin, lung, and brain	Heart and brain	Brain and lungs	Brain and lungs	Lungs	Heart, right lung, brain, and kidney
Age (year)	41	47	45	64	34	50	50	Middle-aged	38
Gender	Female	Male	Male	Male	Female	Female	Female	Female	Male
Condition/malignancy	Stage IV nodular sclerosing Hodgkin's lymphoma	Stage IV A T-lymphoblastic lymphoma	Acute lymphoblastic leukemia	Pre-B acute lymphoblastic leukemia	B-cell acute lymphoblastic leukemia	End-stage kidney diseases due to presumed chronic glomerulonephritis	Hypertension, non-ischemic cardiomyopathy and end-stage renal disease from presumed chronic glomerulonephritis	Leukemia	Autoimmune lymphoproliferative syndrome and secondary hemophagocytic lymphohistiocytosis
Medical intervention	Double umbilical cord blood transplantation	Chemotherapy	Chemotherapy	Chemotherapy	Allogeneic peripheral blood stem cell transplant	Kidney transplant	Kidney transplant	Stem cell transplant	Hematopoietic stem cell transplantation
Exposure	Inhalation of airborne spores of Volvariella *volvacea* (as speculated)					Frequent smelling of fresh *V. volvacea* fruiting bodies	Consumption of *V. volvacea* and their preparation for meals		
Clinical presentation of invasive infection	Patient developed distributive/cardiogenic shock	Fever, headache, slurred speech, and facial droop	Confusion, headache, and macular skin lesions	Fever, confusion, and macular skin lesions	Spiking fever and intermittent headache, acute onset of vertigo; acute onset of diplopia, dysarthria, and loss of consciousness; bilateral subconjuctival hemorrhage; grade 3/6 systolic heart murmur at the apex	Mild headache and 3 weeks of intermittent productive cough and intermittent fever, left-sided facial droop, pronator drift, and sensory neglect	Intermittent fever for 3 weeks, productive cough for 2 weeks, and right-sided headache	Hypotension	Persistent fever
Findings of medical imaging	Chest CT: cavitary lung infarction; wedge-shaped infiltrate in the left upper lobe of the lung; worsening of disease in the left upper lobe. Brain MRI: Focal lesion on the pial surface of the left central sulcus and occipital white matter lesions; progression of the lesions	Pulmonary CT: mass-like consolidation with multiple nodules associated with ground-glass changes. Brain MRI: rim-enhancing mass lesion with perilesional oedema in the lateral aspect of right frontal lobe	Pulmonary CT: focal patchy atelectasis with central cavity in right upper lobe; nodule with central cavitation in left lower lobe. Brain MRI: ring-enhancing nodular lesions in the left cerebral hemisphere and right frontal lobe with variable perilesional oedema and mild left frontal mass effect	Pulmonary CT: lung consolidation predominantly in the right upper and middle lobes with scattered nodular consolidation remaining lung bilaterally. Brain MRI: multiple areas of restricted diffusion with mild, smooth peripheral enhancement in both cerebral hemispheres and cerebellum; small areas of leptomeningeal enhancement	Transesophageal echocardiography: vegetation at mitral leaflet associated with moderate regurgitation. Brain MRI: Acute infarction in the bilateral frontal lobes and right cerebellar hemisphere. Brain CT: acute hemorrhage in the right cerebral hemisphere and pons	Brain MRI: extensive vasogenic oedema in the right parieto-occipital lobe with thrombosed cortical veins. Chest X-ray: right upper zone opacity. Thorax CT: right upper lobe subpleural lesion with cavitation and surrounding glass changes	Chest X-ray: right upper lobe opacity. Thorax CT: right upper lobe nodule. Brain MRI: vasogenic edema in the right parieto-occipital lobe and leptomeningeal enhancement in the right high frontal sulci	Dense consolidation with ground glass opacification in both lungs	Transthoracic echocardiography: vegetation in the left atrium. enhanced CT: multiorgan septic emboli in the brain and kidney. Positron Emission Tomography/CT: left atrial vegetation from the right lung infectious lesion to the left atrium
**Isolation and identification of *V. volvacea***									
Used specimen	Bronchial wash and brain tissue	Brain tissue	Skin tissue	Skin tissue	Mitral valve tissue	Brain tissue	Tissue from right parietal meninges	Bronchoalveolar lavage fluid	Cardiac vegetation and blood plasma
Cell/culture characteristics	Filamentous fungus in bronchial wash and brain biopsy cultures; thin-walled, right-angle-branching septate hyphae (through fungi-fluor stain); hyphae stained positively with Gomori methenamine silver stain, non-sporulating aerial hyphae and substrate hyphae producing oblong to globose thin-walled chlamydospores observed in culture media; chlamydospores matured and eventually showed dolipore septa with a perforated cap		Fungal growth in different culture media	Fungal growth in different culture media	Thin-walled, right-angle-branching hyphae (through Grocott methenamine silver stain)	Straw-colored, flat colony (on Sabouraud dextrose agar supplemented with chloramphenicol) copper-colored, globose to oblong chlamydospores (as shown under microscopy)	Broad septated hyaline hyphae with focal right-angle branching and extensive angioinvasion straw-colored, flat colonies on Sabouraud dextrose agar supplemented with chloramphenicol copper-colored, globose to oblong chlamydospores (shown under microscopy)	Non-sporulating molds (on Sabouraud dextrose agar)	Hyphae-like vegetation removed from the left atrium
Molecular identification	Internal transcribed spacer sequencing	Direct PCR and sequencing of internal transcribed spacer from brain biopsy			Whole genomic DNA with pan fungal PCR	Fungal-specific PCR amplification and internal transcribed spacer sequencing	Fungal-specific PCR amplification and Internal transcribed spacer sequencing	Internal transcribed spacer and D1/D2 sequencing	Plasma metagenomic next-generation sequencing

CT: computed tomography, MRI: magnetic resonance imaging, PCR: polymerase chain reaction

The patients were initially dealing with diseases and had to undergo medical interventions, such as cell/organ transplantation [43,45-49] and chemotherapy [44]. Particularly, the transplant patients were reported to receive conditioning, immunosuppression, and prophylactic treatments to prevent rejection of the transplanted cells or organs, ultimately leading to their immunosuppressed conditions. Subsequently, these patients displayed signs and symptoms of infection affecting their organs, which were also emphasized in imaging results.

*V. volvacea* was characterized by culturing biopsy specimens from the patients on selective media, as well as observing fungal morphologies through staining and microscopy. Molecular methods were also used as definitive confirmation of the fungal infection among the involved organs. Furthermore, different antifungal agents were administered to the patients upon the diagnosis of *V. volvacea* infection as a treatment strategy.

Despite its good reputation for possessing a wide array of functional properties, *V. volvacea* was reported as the etiologic agent of these fungal infections in immunocompromised patients. It is noteworthy that *V. volvacea*, a common edible mushroom, is not part of the roster of fungal species in the human microbiota. This makes fungus rare and/or almost impossible to initiate opportunistic behavior. However, the suppressed immunity of the patients, in addition to potential exposure to the fungal spores, established an opportunity for *V. volvacea* to invade the involved organs, initiating a systemic infection.

### Potential contribution of the biological activities and compounds of Volvariella volvacea to its infective behavior

Relative to the reports of *V. volvacea* infections in immunosuppressed patients, the dual nature of the fungi depicting both the beneficial and harmful effects of their bioactivities, as well as their biocompounds, is the angle being looked into as contributing factors. Since studies have yet to explain the infective nature of the mushroom, these properties were utilized to preliminarily explain the phenomenon.

Two important processes are vital in the pathogenesis of fungal infections: (1) the survival and growth of the pathogens within the host; and (2) the damage inflicted on the host [50]. Possible explanations were provided for the potential contributions of the biological activities and compounds of *V. volvacea* to its infective nature, involving its growth, survival, and virulence within the host. Moreover, the infective tendency of *V. volvacea* was explained based on present reviews and studies highlighting its fellow fungal species that also exhibit pathogenic behavior within their hosts, as well as other physiological processes involved in health and disease.

Generally, some fungi are part of the normal human microbiota. However, these species can act as opportunistic pathogens, especially in immunocompromised hosts [51]. In addition to the reported immunosuppression among patients with *V. volvacea* infection as a predisposing factor and their plausible exposure to the fungal spores, some of the biological activities of the fungus, as listed in Table 2, might also contribute to its infective nature.

**Table 2:** Potential contribution of the bioactivities to the fungal infection

Biological activity	Potential contribution to the *Volvariella volvacea* infection	Supporting principles as described in related studies Summary / findings	Ref.
Antioxidant activity (inhibition of lipid peroxidation)	Serves as a defense mechanism against oxidative stress	*Candida albicans* have developed mechanisms to counter oxidative stress, including lipid peroxidation, to survive and proliferate within its host.	[55]
Antioxidant activity (inhibition of tyrosinase)	Serves as a strategy to outgrow normal microbiota and other pathogens within the host	Literature review revealed that natural and synthetic tyrosinase inhibitors, in addition to their tyrosinase inhibitory activity, also have antibacterial activity.	[59]
Antioxidant activity (reduction of ferric ions and ferrous iron chelating activity)	Helps acquiring iron from within the host, initiating a competition	Literature review highlighted iron uptake strategies of fungi, including reductive pathways (through their ferric reductases) and biosynthesis of iron-chelating siderophores.	[57]
		*Candida albicans* acquires heme using ferric reductase-related protein.	[58]
Antioxidant activity (increased superoxide dismutase)	Serves as a defense mechanism against oxidative stress	Fungal pathogen *Paracoccidioides* utilizes superoxide dismutase as a defense mechanism against reactive oxygen species within the host.	[52]
		Literature review highlighted that superoxide dismutase activity expression at the cell surface of *Candida albicans* is triggered by reactive oxygen species.	[53]
Antioxidant activity (increased catalase)	Serves as a defense mechanism against oxidative stress	Increased catalase enhances the fitness of *Candida albicans* in the presence of oxidative stress.	[54]
Antioxidant activity (increased glutathione peroxidase)	Serves as a defense mechanism against oxidative stress	Dimorphic fungus *Talaromyces marneffei* regulates genes encoding enzymes, including glutathione peroxidase to use glutathione for stress responses and morphological adaptability.	[56]
Cytotoxic and cell inhibitory activity (reduction and inhibition of some cell lines)	Contributes to the damage of host cells and tissues	Fungal pathogens play a role in cell death during host-pathogen interactions via apoptosis, necrosis, necroptosis, pyroptosis, and ETosis.	[67]
Immunomodulatory activity (increased generation of CD11c+BMDC [through *Volvariella volvacea*’s bioactive compound, FIP-vvo])	Contributes to inflammation	Increased CD11c+BMDC contributed to inflammation, fibrosis, and hypertrophy in the left ventricle.	[60]
Immunomodulatory activity (activation of MAPK signaling pathway in RAW264.7 cells [through *Volvariella volvacea* bioactive compound, VGPI-a])	Promotes tumor progression	Amplification of MAPK signaling pathway promoted lung adenocarcinoma progression, specifically in the absence of p53 gene expression.	[61]
Immunomodulatory activity (promotion of pro-inflammatory genes)	Promotes its survival and growth	A type of inflammatory bowel disease favored fungal growth at the expense of bacteria.	[62]
		A type of inflammatory bowel disease disrupted production of sIgA antibodies against *Candida albicans*.	[63]
	Contributes to the damage of host cells and tissues	Elevation of pro-inflammatory molecules can lead to hyper-inflammation, damaging tissues and contribute to the development of infection, as well as diseases.	[64-66]
Antimicrobial activity (bacterial inhibition)	Helps competing and/or inhibiting bacterial species present within the host	Fungal pathogen *Aspergillus fumigatus* inhibited *Pseudomonas aeruginosa* in cystic fibrosis airways.	[69]
		A review of reports emphasized competition between fungal and bacterial pathogens for nutrients and adhesion sites within their host.	[70]
Inhibition of platelet aggregation and platelet aggregating factor	Helps evading the defense mechanism of the host body to induce platelet aggregation during infection	The inefficient platelet activity/reaction in the presence of *Candida* may favor the survival of the fungus when present in blood.	[71]

MAPK: mitogen-activated protein kinase, BMDC: bone marrow-derived dendritic cells

### Potential infective mechanisms related to antioxidant activity

Some antioxidant mechanisms of *V. volvacea*, including lipid peroxidation inhibition [15, 20] and increased superoxide dismutase, catalase, and glutathione peroxidase [22], may all contribute to the growth and survival of the fungi, especially as defense mechanisms against oxidative stress. This corresponds with the proliferation and survival tactics of *Paracoccidioides* [52] and *Candida albicans* [53-55] within their host tissues, effectively evading oxidation. Meanwhile, the utilization of glutathione metabolism through gene encoding regulation of glutathione peroxidase helps *Talaromyces marneffei* to morphologically adapt and respond to stress, as well as to evade macrophage killing [56].

Reductive pathways and biosynthesis of iron-chelating siderophores are some of the uptake strategies of fungi to acquire iron for growth [57]. In addition, Roy et al. [58] in their study revealed that *C. albicans* can acquire heme using ferric reductase-related proteins. This can suggest that the ability of *V. volvacea* to reduce ferric ions [13-17,19] and chelate ferrous iron [23] helps the fungus compete with its host for iron acquisition. Regarding this competition, Yuan et al. [59] highlighted the relationship between tyrosinase-inhibitory and antibacterial activities of tyrosinase inhibitors. This may suggest that *V. volvacea* inhibits tyrosinase as a strategy to outgrow and/or compete with the normal microbiota and other pathogens during its invasion within the host tissues.

Overall, the antioxidant activity of *V. volvacea* may assist it in evading the host defense mechanisms during an infection, such as induction of oxidation. This allows the fungus to survive and proliferate within tissues in spite of the immune response of the host. In addition to the antioxidant mechanisms of the fungus, the reduction of ferric iron and chelating activity may be used by *V. volvacea* as uptake strategies in acquiring iron from within the host, initiating a competition.

### Potential infective mechanisms related to immunomodulatory activity

Increased generation of bone marrow-derived dendritic cells (BMDCs) [24] and activation of the mitogen-activated protein kinase (MAPK) signaling pathway [25] exhibited by the bioactive compounds of *V. volvacea*, namely FIP-vvo and VGPI-a, respectively, are advantageous in developing immunomodulatory agents. However, as revealed in the study of Wang et al. [60], increased BMDCs contributed to inflammation, fibrosis, and hypertrophy in the left ventricle. Moreover, the promotion of lung adenocarcinoma progression was the reported negative effect of the amplified MAPK signaling pathway by Cicchini et al. [61]. Collectively, these two immunomodulatory mechanisms can likely be additions to the biological activities that contribute to the detrimental effects of *V. volvacea* infection.

An inflammatory environment favors fungal growth at the expense of bacteria in patients with Crohn's disease, a type of inflammatory bowel disease [62]. Correspondingly, the same type of inflammatory bowel disease disrupted the production of sIgA antibodies that target *C. albicans* [63]. This may explain how the promotion of pro-inflammatory genes by *V. volvacea* may aid its growth and survival, exacerbating the already dangerous inflammation within the host tissues. Most especially, the elevation of pro-inflammatory molecules can lead to hyper-inflammation, tissue damage, and infection development [64-66].

The immunomodulatory activity of *V. volvacea*, together with its cytotoxicity, additionally confers growth and survival of the fungus. But most especially, these activities may influence the pathogenicity and virulence of the fungus, serving as a damaging contributor within the host tissues and cells.

### Potential infective mechanisms related to other biological activities

The cytotoxic, antimicrobial, and antiplatelet activities exhibited by *V. volvacea* may also be factors conducive to the invasive and infective tendency of the fungus. Similar to antioxidant and immunomodulatory mechanisms, the three bioactivities may also be utilized by the fungus for growing, surviving, and proliferating within its host.

One of the key virulent factors of fungal infections is cell death induction through different mechanisms that were highlighted in the review of Camilli et al. [67]. These mechanisms are apoptosis, necrosis, necroptosis, pyroptosis, and ETosis. This is consistent with a study performed by Mihn et al. [26], which revealed the capability of *V. volvacea* to induce cell death via apoptosis. While the inhibited cells in the study were Hep-3B cancer cell lines, it should be noted that the activity of any cytotoxic agents can be non-specific, killing both harmful and healthy cells within the host. The most applicable utilization of cytotoxic agents, which is chemotherapy used in treating cancers and tumors, is also notorious for damaging even the dividing normal cells, like bone marrow cells, cell linings in the reproductive and gastrointestinal tracts, and hair follicle cells [68]. Chen et al. [28] also reported the inhibitory activity of *V. volvacea* against HepG-2 and PC-3M cancer cell lines.

Through dysbiosis, which is the imbalance in the diversity of normal microbiota in the human body, fungal species can switch to their opportunistic nature and begin invading the tissues within the host. With the antimicrobial activity of *V. volvacea* [33-36, 39], the fungus may inhibit both beneficial and harmful bacteria as it becomes invasive and infective. Similarly, Reece et al. noticed the same occurrence with *Aspergillus fumigatus* against *Pseudomonas aeruginosa* [69], as the fungal species was observed to inhibit the bacteria in cystic fibrosis airways. Furthermore, the competition for nutrients and adhesion sites between fungal and bacterial pathogens within tissues [70] suggests that *V. volvacea* can display an antagonistic behavior against other bacteria and even its fellow fungi. This can potentially result in dysbiosis, which may later induce serious health problems, like infection and diseases.

Moreover, the bacterial inhibition capacity of *V. volvacea*, along with tyrosinase inhibition [59], may serve as a tactic for the fungus to outgrow both beneficial and pathogenic microorganisms in the host microbiota. This mechanism paves the way for the fungus to eliminate microbial competitors, making the invasion of the host tissues and establishment of infection more effective.

Moreover, as an evading tactic, the antiplatelet activity of *V. volvacea* can help the fungus proliferate in the bloodstream. Through its inhibition of platelet-aggregating factor and platelet aggregation [27], *V. volvacea* may evade the defense mechanisms of the host body to initiate platelet aggregation during infection. This is in line with the findings of a study performed by Eberl et al. [71] revealing an inefficient platelet activity in the presence of *Candida*. This signifies the favorable survival condition of the fungus when present in blood.

### Potential infective mechanisms related to biological compounds

Compounds of *V. volvacea* are generally linked to its biological activities, which were previously utilized to potentially explain the pathogenic nature of the fungus. While these compounds have corresponding influences on biological activities, present studies and reviews highlighting them may provide additional insight into the detrimental effects of the fungus during its infection in immunocompromised hosts. The most reported biological compounds of *V. volvacea* include phenols [10, 11, 13, 14, 16-20, 31], flavonoids [10, 14, 16, 18, 34] , and β-glucan [14, 15, 19]. Their additional contribution to the infective nature of the fungus is outlined in Table 3.

**Table 3:** Potential contribution of the biological compounds to the infection

Most reported compounds of *Volvariella volvacea*	Potential contribution to the infection	Supporting principles as described in related studies	
		Summary / findings	Reference
Phenols	May induce toxicity and detrimental effects within the host cells and tissues when overabundant	Can act as pro-oxidants, causing oxidation and DNA damage by producing free radicals;	[72-73]
		Exhibits toxicity, mutagenesis, and carcinogenesis	[75]
Flavonoids	May induce toxicity and detrimental effects within the host cells and tissues when overabundant	Can act as pro-oxidants, causing oxidation and DNA damage by producing free radicals;	[72, 74]
		Can induce upset stomach and headaches as side effects; may generate byproducts that cause loss of protein function; May decrease the activity of the immune system;	[77]
		Exhibits carcinogenicity, mutagenicity, and toxicity in liver and kidney	[76]
β-glucan	Contributes to growth, survival, and virulence	Confers structural integrity to the cell wall;	[78]
		Contributes to the adherence of fungal biofilm, structural integrity, and drug resistance	[79]

Among the three compounds, phenols and flavonoids, which are known to be antioxidants, can also exhibit pro-oxidant activities, causing oxidation and cell damage [72-74]. Both can also exhibit toxic effects, like carcinogenicity and mutagenicity to cells [75-76]. Furthermore, protein loss and immunosuppression may also be induced by flavonoids, along with upset stomach and headaches as side effects [77].

Meanwhile, as β-glucan confers the structural activity of fungal cell walls [78], it may also contribute to the growth, survival, and virulence of opportunistic fungal pathogens. The polysaccharide is also utilized by fungal species in biofilm formation. In addition, β-glucan is linked to the adherence, structural integrity, and drug resistance of the biofilm [79], effectively aiding the survival of any opportunistic fungi, including *V. volvacea*, within its host during infection.

Overall, the biological compounds may contribute to the infection through their linkage with the biological activities of the fungus. This is especially because these compounds themselves also exhibit the aforementioned functional mechanisms, in addition to their toxicological properties.

## Conclusion

This review aimed to present cases of systemic infection in immunocompromised hosts caused by *V. volvacea*. Aside from the immunosuppression of the patients as a predisposing factor and their potential exposure to fungal spores, the reported biological activities and compounds of *V. volvacea* were considered contributing factors that may preliminarily explain its infective nature in this bifaceted review. These functionalities and properties of *V. volvacea*, although deemed beneficial, may contrastingly contribute to the growth, survival, proliferation, and virulence of the fungus as it induces infection. The explication was based on related studies describing the infective mechanisms of opportunistic and true pathogenic fungal species, as well as other biological processes involved in health and diseases. Figure 1 illustrates the framework for how these bioactivities and compounds may contribute to the opportunistic infection of *V. volvacea*.

**Figure 1:**
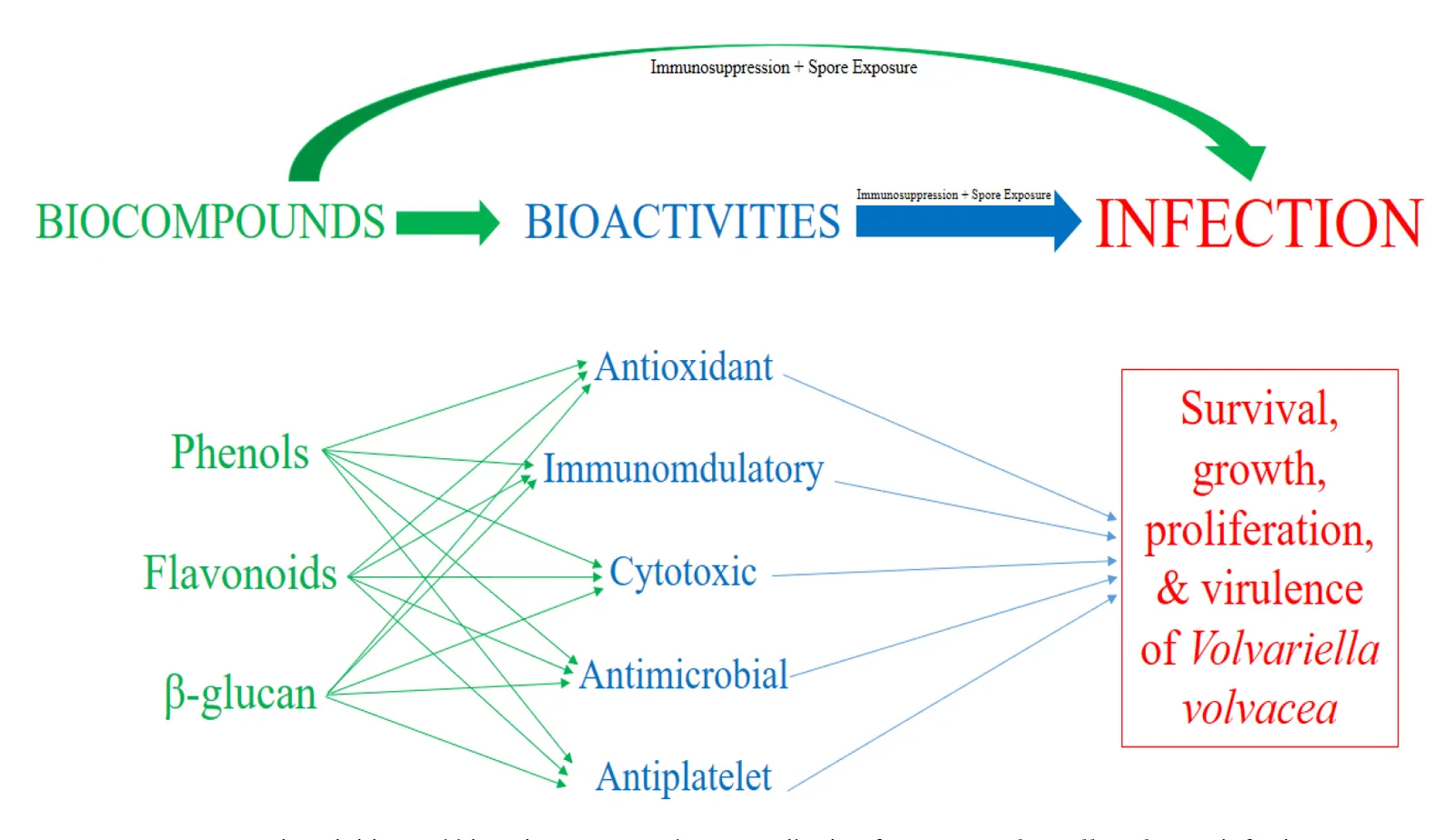
Bioactivities and bioactive compounds as contributing factors to *Volvariella volvacea* infection

Meanwhile, even if the mechanism of infection is yet to be fully understood, it is strongly advised to exercise utmost precautionary and biosafety measures when handling *V. volvacea*. To reduce the potential exposure to fungal spores in a laboratory, production, or kitchen setting, it is critical to wear personal protective equipment, such as a laboratory gown, gloves, face mask, and goggles. Furthermore, maintaining overall health is vital, particularly for people who are frequently exposed to mushrooms, as a protection from any opportunistic behavior that may be exhibited by *V. volvacea*.
